# Pancreaticoduodenectomy with Preservation of a Prior Hepaticojejunostomy for Ampullary Carcinoma: A Case Report

**DOI:** 10.70352/scrj.cr.26-0186

**Published:** 2026-06-10

**Authors:** Yasunori Uesato, Susumu Inamine

**Affiliations:** Department of Metabolic Surgery, Ohama Daiichi Hospital, Naha, Okinawa, Japan

**Keywords:** ampullary carcinoma, pancreaticoduodenectomy, hepaticojejunostomy, reoperation, reconstruction

## Abstract

**INTRODUCTION:**

Pancreaticoduodenectomy (PD) can be technically challenging in patients who previously underwent biliary reconstruction, particularly hepaticojejunostomy. The optimal strategy for managing a prior hepaticojejunostomy during PD has not been well established.

**CASE PRESENTATION:**

A woman in her 70s developed ampullary carcinoma 10 years after diversion surgery for pancreaticobiliary maljunction. She had previously undergone laparoscopic extrahepatic bile duct resection with hepaticojejunostomy. Subtotal stomach-preserving pancreaticoduodenectomy was planned. Intraoperatively, dense adhesions were observed around the hepaticojejunostomy, and the previous bile duct transection had been performed near the hepatic duct bifurcation. Resection of the existing anastomosis was considered technically risky because it might have resulted in multiple small biliary openings and increased operative complexity. Therefore, the hepaticojejunostomy was preserved based on intraoperative findings. Reconstruction was completed using a single jejunal limb for pancreaticojejunostomy and gastrojejunostomy. The operative time was 430 min, and blood loss was 192 mL. The postoperative course was uneventful. Pathological examination revealed well-differentiated tubular adenocarcinoma of the ampulla of Vater (pT1aN0M0, Stage IA) with negative margins. The patient remains recurrence-free 6 months after surgery.

**CONCLUSIONS:**

Careful intraoperative assessment is important when determining whether a prior hepaticojejunostomy should be preserved during PD. Preservation may represent a reasonable option when re-dissection around the hepatic hilum is technically hazardous.

## Abbreviations

ERCP
: endoscopic retrograde cholangiopancreatography
PBM
: pancreaticobiliary maljunction
PD
: pancreaticoduodenectomy
SSPD
: subtotal stomach-preserving pancreaticoduodenectomy

## INTRODUCTION

Carcinoma of the ampulla of Vater is a relatively uncommon malignancy arising from the periampullary region.^1)^ PD, including SSPPD, remains the standard curative treatment.^2)^ Achieving complete resection with negative margins is essential for long-term survival.

In patients who have previously undergone biliary reconstruction, PD can be technically challenging. Hepaticojejunostomy alters the normal anatomy of the hepatic hilum and is often associated with dense adhesions. During reoperation, management of the existing biliary-enteric anastomosis becomes a critical issue. However, the optimal strategy for handling a prior hepaticojejunostomy during PD has not been clearly established because of the extremely limited number of reported cases.

PBM is a congenital anomaly characterized by an abnormal union of the pancreatic and bile ducts and is associated with the development of biliary tract malignancies.^3)^ Diversion surgery, including extrahepatic bile duct resection with hepaticojejunostomy, is generally recommended to reduce this risk. Although carcinoma arising in the residual bile duct has been reported after diversion surgery, the occurrence of ampullary carcinoma in this setting appears to be uncommon.^4)^

Here, we report a case of ampullary carcinoma that developed 10 years after diversion surgery for PBM. In this patient, SSPPD was performed with preservation of the previous hepaticojejunostomy. We describe the intraoperative decision-making process and discuss reconstructive options in patients undergoing PD after prior biliary reconstruction.

## CASE PRESENTATION

A woman in her 70s had undergone laparoscopic extrahepatic bile duct resection and hepaticojejunostomy for PBM 10 years earlier. At the previous surgery, the bile duct had been transected near the hepatic duct bifurcation.

Three months before referral to our department, she developed acute cholangitis with abscess formation in the posterior segment of the liver. ERCP was performed, and a biliary stent was placed. During ERCP, a mass was detected at the ampulla of Vater, and biopsy revealed adenocarcinoma.

Contrast-enhanced CT showed an approximately 20-mm lesion at the ampulla without distant metastasis. The clinical diagnosis was ampullary carcinoma, cStage I. SSPPD was planned.

Preoperatively, we considered 3 reconstructive strategies: (1) resection of the previous hepaticojejunostomy with redo biliary reconstruction, (2) preservation of the hepaticojejunostomy with reconstruction using a newly elevated jejunal limb, and (3) preservation of the hepaticojejunostomy with completion of reconstruction using a single jejunal limb.

**Fig. 1:**
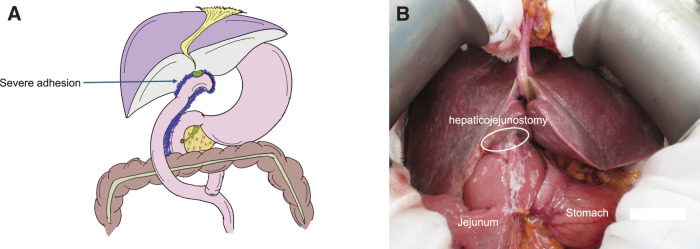
Intraoperative findings showing severe adhesions around the prior hepaticojejunostomy. (**A**) Schematic illustration of the operative field demonstrating dense adhesions around the hepaticojejunostomy and the adjacent jejunal mesentery. (**B**) Intraoperative photograph demonstrating the hepaticojejunostomy located beneath the liver.

The surgery was performed through an upper midline abdominal incision. Dense adhesions were found around the previous hepaticojejunostomy (**Fig. 1**). In addition, dense adhesions were also observed between the elevated jejunal mesentery and the duodenum, requiring careful dissection to avoid mesenteric injury. These adhesions were difficult to dissect and were considered to carry a high risk of bleeding. In addition, because the previous bile duct transection had been performed near the hepatic duct bifurcation, further resection was expected to result in 2 separate hepatic duct orifices. Standard regional lymph node dissection for PD was performed. Reconstruction under such conditions was considered technically demanding. Therefore, we decided to preserve the existing hepaticojejunostomy.

**Fig. 2:**
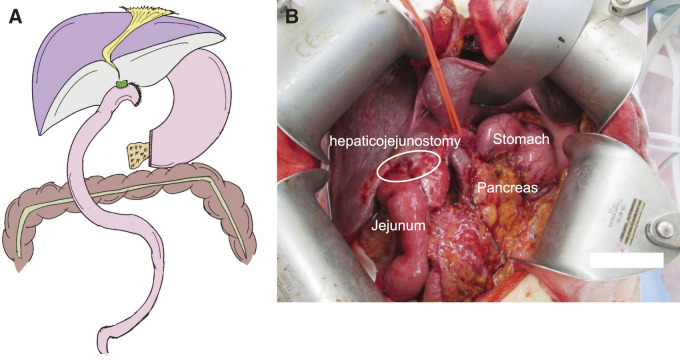
Operative field after PD with preservation of the hepaticojejunostomy. (**A**) Schematic illustration showing the operative configuration after removal of the pancreatic head and duodenum while preserving the hepaticojejunostomy. (**B**) Intraoperative photograph demonstrating the preserved hepaticojejunostomy adjacent to the pancreatic remnant. PD, pancreaticoduodenectomy

**Fig. 3:**
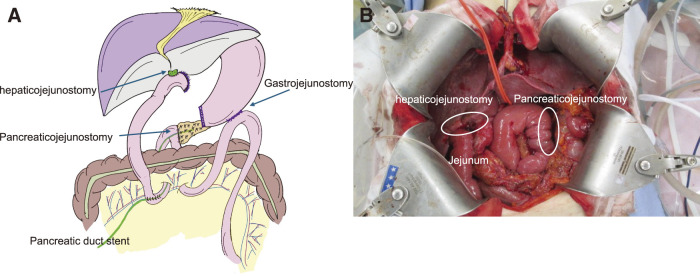
Reconstruction using a single jejunal limb with preservation of the hepaticojejunostomy. (**A**) Schematic illustration of the reconstruction. The pancreatic remnant was anastomosed to the jejunum 40 cm distal to the hepaticojejunostomy, and a gastrojejunostomy was performed 30 cm distal to the pancreaticojejunostomy using the same jejunal limb. (**B**) Intraoperative photograph demonstrating the preserved hepaticojejunostomy and pancreaticojejunostomy.

After resection of the pancreatic head and duodenum, the existing hepaticojejunostomy was left intact (**Fig. 2**). Reconstruction was then completed using a single jejunal limb (**Fig. 3**). The preserved jejunal limb was carefully evaluated intraoperatively for adequate blood supply based on serosal color, absence of congestion, and preserved peristalsis. Particular attention was paid to preserving mesenteric orientation and preventing torsion throughout reconstruction. A jejunal segment located 40 cm distal to the hepaticojejunostomy was elevated via a retrocolic route for pancreaticojejunostomy, because minimizing tension and anatomical mismatch at the pancreaticojejunostomy site was considered particularly important to reduce the risk of pancreatic leakage. Then, 30 cm distal to the pancreaticojejunostomy, the jejunum was elevated via an antecolic route for gastrojejunostomy. Braun anastomosis was not performed.

Because postoperative endoscopic removal was considered difficult, the biliary stent was removed intraoperatively through a small enterotomy made on the anterior wall of the jejunum near the hepaticojejunostomy.

The operative time was 430 min, and blood loss was 192 mL. No clinically relevant postoperative pancreatic fistula or other major complications were observed. The patient was discharged on POD 10.

Histopathological examination revealed a well-differentiated tubular adenocarcinoma (tub1), flat-expanding type, measuring 20 × 10 mm. The tumor was confined to the mucosa (pT1a [M]) without lymphatic, venous, or perineural invasion (ly0, v0, ne0). No lymph node metastasis was identified (pN0). All resection margins were negative (R0; HM0, PM0, EM0). The final pathological stage was pStage IA.

The patient remains free of recurrence 6 months after surgery.

## DISCUSSION

PD is the standard surgical treatment for carcinoma of the ampulla of Vater. However, PD in patients with previous biliary reconstruction can be technically challenging because prior surgery often results in dense adhesions and altered anatomy around the hepatic hilum. In such situations, management of the existing biliary-enteric anastomosis becomes an important intraoperative consideration. Only a very limited number of reports have described PD after prior hepaticojejunostomy.^5)^ Most of these reports are isolated case reports, and no study has systematically compared reconstructive strategies, including preservation versus resection of the existing hepaticojejunostomy, or established clear criteria for selecting among these approaches. Prior hepaticojejunostomy often causes dense adhesions around the hepatic hilum and changes the normal anatomy.^6)^

A key issue in this situation is how to manage the existing biliary-enteric anastomosis. Dissection around the hepatic hilum may result in significant bleeding because of severe adhesions.^7)^ In the present patient, the bile duct had been divided close to the confluence of the right and left hepatic ducts during the previous surgery. Resection of the existing hepaticojejunostomy might therefore have resulted in 2 small biliary openings, which would have required technically demanding biliary reconstruction.

**Fig. 4:**
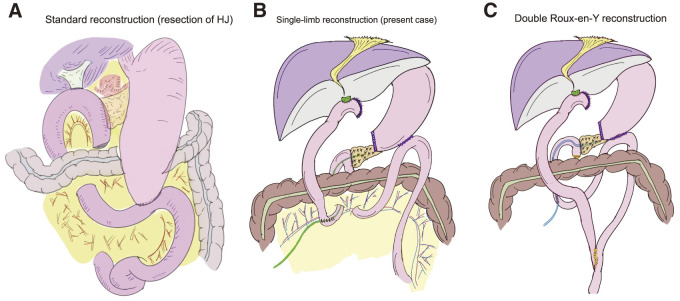
Reconstructive options for PD after prior hepaticojejunostomy. (**A**) Standard reconstruction after resection of the previous hepaticojejunostomy. (**B**) Reconstruction using a single jejunal limb with preservation of the hepaticojejunostomy (present case). (**C**) Reconstruction using a double Roux-en-Y configuration while preserving the hepaticojejunostomy. HJ, hepaticojejunostomy; PD, pancreaticoduodenectomy

In general, several reconstructive options may be considered in patients undergoing PD after prior hepaticojejunostomy (**Fig. 4**). First, the previous hepaticojejunostomy can be resected and a new biliary reconstruction performed as part of standard PD. Although this approach restores the usual anatomy, it requires extensive dissection around the hepatic hilum. Second, the hepaticojejunostomy can be preserved, and reconstruction can be completed using a newly elevated jejunal limb. While this approach allows the use of a fresh jejunum, management of multiple jejunal limbs may increase the risk of mesenteric twisting or tension. Third, as performed in the present case, reconstruction can be completed using a single jejunal limb while preserving the existing hepaticojejunostomy. A review of the available literature suggests that PD after prior hepaticojejunostomy is extremely uncommon, and currently available evidence is limited to a small number of case reports. Furthermore, these reports primarily describe technical feasibility in individual cases rather than providing comparative evaluation of reconstructive strategies or long-term outcomes. To our knowledge, no previous study has specifically examined how preservation versus resection of a prior hepaticojejunostomy should be selected according to intraoperative findings. Therefore, surgical decision-making in this setting remains largely individualized, and practical guidance based on accumulated evidence is lacking.

The clinical significance of the present case lies not in the novelty of the reconstructive technique itself, but in clarifying the conditions under which preservation of a prior hepaticojejunostomy may be a reasonable option during PD. Although preservation of the existing anastomosis may appear to be an intuitive decision, clear criteria for selecting this strategy over resection and redo biliary reconstruction have not been well established in the literature. From our experience, preservation may be particularly appropriate in situations where re-dissection around the hepatic hilum is technically hazardous due to dense adhesions, where prior bile duct transection was performed near the hepatic duct bifurcation and further resection would result in multiple small biliary openings, and where additional biliary resection is not required from an oncological standpoint. Under such conditions, preservation of the existing hepaticojejunostomy may help avoid complex biliary reconstruction and reduce operative risk.

In this patient, we chose to preserve the hepaticojejunostomy based on intraoperative findings. Severe adhesions were observed around the anastomotic site, and further dissection was considered unsafe. Preservation of the hepaticojejunostomy allowed us to avoid extensive hilar dissection and complex biliary re-anastomosis. In addition, reconstruction using a single jejunal limb was designed to simplify the reconstructive procedure while prioritizing the safety of the pancreaticojejunostomy, which was considered the most critical anastomosis because of the potentially severe consequences of postoperative leakage. Therefore, particular attention was paid to preserving jejunal blood flow through intraoperative assessment of bowel color, venous congestion, and peristalsis, as well as to maintaining mesenteric orientation and minimizing tension to avoid torsion. A Braun anastomosis was not added because intestinal passage was considered adequate, and minimizing additional anastomoses was prioritized to avoid unnecessary procedural complexity. However, this approach may still have potential disadvantages, including impaired jejunal blood flow or mesenteric torsion, and careful intraoperative judgment remains essential.

Pathological examination showed pT1a disease with negative margins and no lymph node metastasis.^8)^ In this early-stage case, preservation of the hepaticojejunostomy did not appear to compromise curative resection. However, this finding should be interpreted with caution, and the oncological safety of this approach cannot be generalized to more advanced cases, particularly those requiring additional biliary resection.

PBM is known to be associated with biliary tract malignancies, particularly gallbladder and bile duct cancer. However, the relationship between PBM and ampullary carcinoma remains unclear. In the present case, a causal association between PBM and the development of ampullary carcinoma cannot be determined.

This report has several limitations. It describes only 1 case, and the follow-up period is short. Further experience is required to clarify the most appropriate surgical strategy in similar patients.

## CONCLUSIONS

In such situations, careful intraoperative assessment and individualized decision-making are essential when determining whether the existing hepaticojejunostomy should be preserved or reconstructed. In selected patients, preservation of a prior hepaticojejunostomy may represent a reasonable reconstructive option when re-dissection around the hepatic hilum is technically hazardous and oncological compromise is unlikely.

## DECLARATIONS

### Availability of data and materials

The datasets supporting the conclusions of this article are included within the article.
